# Screening for C3 Deficiency in Newborns Using Microarrays

**DOI:** 10.1371/journal.pone.0005321

**Published:** 2009-04-24

**Authors:** Magdalena Janzi, Ronald Sjöberg, Jinghong Wan, Björn Fischler, Ulrika von Döbeln, Lourdes Isaac, Peter Nilsson, Lennart Hammarström

**Affiliations:** 1 Division of Clinical Immunology, Karolinska Institutet, KUS Huddinge, Stockholm, Sweden; 2 Department of Proteomics, School of Biotechnology, AlbaNova University Center, KTH-Royal Institute of Technology, Stockholm, Sweden; 3 Children's Hospital, KUS Huddinge, Stockholm, Sweden; 4 Centre for Inherited Metabolic Diseases, Karolinska Institutet, KUS Huddinge, Stockholm, Sweden; 5 Laboratory of Complement, Department of Immunology, Institute of Biomedical Sciences, University of São Paulo, São Paulo, Brazil; Charité-Universitätsmedizin Berlin, Germany

## Abstract

**Background:**

Dried blood spot samples (DBSS) from newborns are widely used in neonatal screening for selected metabolic diseases and diagnostic possibilities for additional disorders are continuously being evaluated. Primary immunodeficiency disorders comprise a group of more than one hundred diseases, several of which are fatal early in life. Yet, a majority of the patients are not diagnosed due to lack of high-throughput screening methods.

**Methodology/Principal Findings:**

We have previously developed a system using reverse phase protein microarrays for analysis of IgA levels in serum samples. In this study, we extended the applicability of the method to include determination of complement component C3 levels in eluates from DBSS collected at birth. Normal levels of C3 were readily detected in 269 DBSS from healthy newborns, while no C3 was detected in sera and DBSS from C3 deficient patients.

**Conclusions/Significance:**

The findings suggest that patients with deficiencies of specific serum proteins can be identified by analysis of DBSS using reverse phase protein microarrays.

## Introduction

Primary immunodeficiency disorders (PIDs) constitute a group of inherited defects of the immune system with an estimated prevalence of one in 500 in the United States [1]. Many of the affected individuals are asymptomatic, but more severe defects may result in a fatal outcome if untreated. Some patients show an increased susceptibility to infections during the first years of life, yet, a majority of them are not diagnosed during childhood due to lack of suitable analytical methods. As many of the acquired infections are severe, early diagnosis might prevent organ damage or even death.

Defects of the complement system constitute one of the vastly under-diagnosed PID groups [2]. The third component of complement (C3) is the most abundant protein in the complement system with serum levels of approximately 1 g/l in both adults and newborns. It plays an important role in the clearing of infections as it is involved in cytolysis, increased vasopermeability, opsonization, clearance of immune complexes and facilitation of B cell proliferation and differentiation. Individuals lacking C3 are therefore highly susceptible to recurrent systemic bacterial infections and frequently suffer from immune complex-related disorders, such as glomerulonephritis and systemic lupus erythematosus [3]. The deficiency is caused by mutations in the *C3* gene, resulting in absence/markedly reduced levels, or dysfunction of the protein [4]. The disorder is rare, only 23 families (31 individuals) have been identified to date [for review see 3], [5]–[7] and ten different, unique mutations have been found in the *C3* encoding gene [8]–[15]. In most cases, the defect is found in consanguineous family and noted already in infancy or early childhood due to life-threatening infections during the first year of life. Current complement analysis in clinical practice is restricted to cases where a patient's health status is brought to attention, often due to severe infections. Thus, an early diagnosis of complement deficiency may decrease the risk of acquiring severe infections during childhood by using prophylactic treatment.

In the 60s, neonatal screening programs for metabolic disorders, such as phenylketonuria, based on the use of dried blood spot samples (DBSS), were introduced in several countries. Today, they constitute an established part of the neonatal healthcare system. In Sweden, DBSS have been collected from all newborns and stored since 1975. As reviewed by McDade *et al*, 2007, there is a growing interest in the use of DBSS in large population-based biomarker studies; the study of Tsunami Aftermath and Recovery being the largest to date with 35 000 DBSS collected [16]. The DBSS have also been used in multiplex analysis of inflammatory markers [17] and in 2008, the Jeffrey Modell Foundation and the State of Wisconsin launched a DBSS based newborn screening program for severe combined immunodeficiency [18]. During 2008, all children born in Wisconsin have been screened and it is anticipated that several additional states will join the pilot screening program during 2009.

In the present study, we investigated whether patients with C3 deficiency can be identified by the analysis of DBSS eluates using reverse phase serum microarrays. This assay, previously described by Janzi *et al*, 2005, provides a novel platform suitable for large-scale, semi-quantitative determination of concentrations of serum proteins [19].

## Results

### C3 levels in serum samples

**Figure 1:**
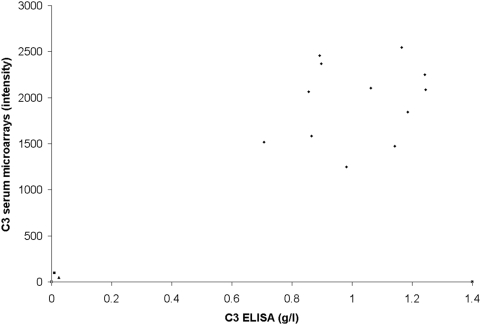
Correlation between serum microarrays and ELISA for C3 levels in serum samples. The correlation between serum microarray intensities (arbitrary units) and C3 concentration (g/l) for serum samples from 12 controls (circles), Swedish C3 deficient patient (triangle) and Brazilian C3 deficient patient (square).

C3 deficient sera from two non-related patients were used in this study. Serum samples from twelve controls and the Swedish [20] and Brazilian patients [21] were analysed for C3 levels by ELISA and serum microarrays. No C3 was detected in the two C3 deficient patient samples, whereas both methods readily detected C3 in all twelve control serum samples (varying from 0.7 to 1.2 g/l), with a correlation of 0.83 (Figure 1).

### Determination of elution procedure for DBSS

To determine the appropriate temperature and time of elution for proteins from the DBSS, different conditions were tested using 12 simulated DBSS, prepared using a freshly collected adult blood control sample containing 1.3 g/l. Each constructed DBSS contained 15 µl of blood (approximately 9 µl of plasma) and the samples were eluted in 4°C, room temperature, or 37°C, for one hour, 24 hours, 48 hours or one week. The C3 levels were determined by ELISA and a ratio between the original amounts of C3 in the blood sample and the amounts obtained from the DBSS, was calculated. The amount of C3 obtained when testing various conditions ranged from 0.9 to 1.3 g/l, giving a ratio from 3∶5 (37°C, one hour) to 1∶1 (4°C, one week). To ensure effective elution, the DBSS included in the study were thus eluted for one week at 4°C.

### C3 levels in DBSS

**Figure 2:**
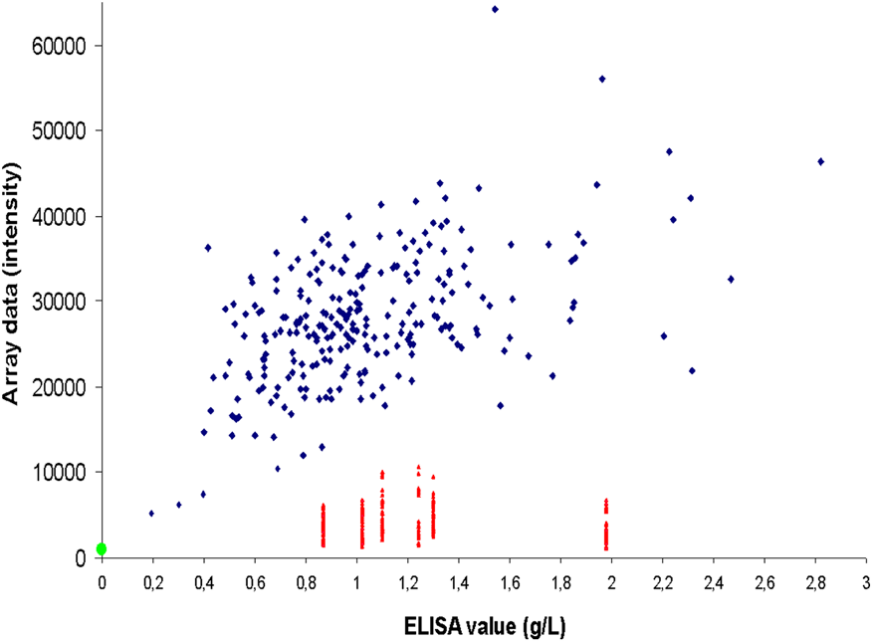
Correlation between serum microarrays and ELISA for C3 levels in DBSS. The correlation between serum microarray intensities and ELISA values for the Swedish C3 deficient patient (green circle) and 269 controls. Six samples (marked in red) show low intensities on the microarrays when compared to C3 concentration (g/l). The results for the low intensity samples are based on 6 printings and a total of 20–47 replicates.

**Figure 3:**
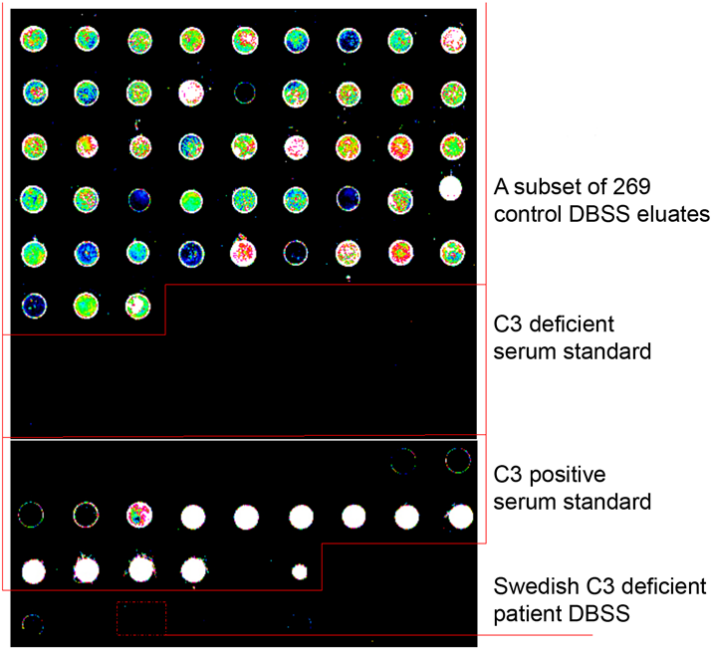
Visualization of the fluorescence intensitites of the microarrays. The upper part shows a subset of the 269 control DBSS. The Swedish C3 deficient and C3 positive serum standards contains 23 serial dilutions each (high to low dilution, 1∶5 to 1∶100 000). The two lowest dilutions of the standard curve are separated by a blank (PBS). The dotted rectangle depicts the C3 deficient sample.

The storage time for the DBSS prior to elution and analysis was 20 years for the Swedish patient and 8–23 years for thirteen of the controls. The remaining control DBSS had not been stored before analysis. No C3 was detected in the DBSS eluate from the Swedish patient using neither ELISA nor microarrays. In the control group (n = 269), C3 was readily detected in all samples, with a correlation between the two methods of 0.49 (Figure 2). The mean C3 level of 0.6 g/l in the 13 stored controls was significantly lower (P<0.01) than the mean level of 1.0 g/l in the freshly collected controls. The levels of C3 in both control groups were significantly higher (P<0.01) than in the C3 deficient patient sample, indicating that both methods are suitable for identification of C3 deficiency using DBSS eluates. The morphology of the microarray spots was similar to those previously described [19] (Figure 3).

As no original DBSS was available from the Brazilian C3D patient, a simulated DBSS was created by mixing serum from the Brazilian patient and blood cells from the Swedish C3D patient. Positive and negative controls were prepared in the same manner. Simulated DBSS have been used in previous studies on PIDs [22]. Similar to the original DBSS of the Swedish patient, no C3 was detected in the Brazilian DBSS or negative controls, while all 9 positive controls had detectable levels of C3 (P<0.01), using both ELISA and serum microarrays (data not shown).

To determine the coefficient of variation (CV) for measuring C3 levels in DBSS using ELISA, we tested the C3 levels in 14 randomly selected DBSS from our collection of control samples at four different occasions. The CV was 29±13%, which was significantly higher than that of the microarrays (15±6%).

### Re-analysis of DBSS with low C3 levels

Six control DBSS showed consistently low intensities on serum microarrays but normal C3 levels on ELISA. The samples were therefore re-printed together with the DBSS eluate of the Swedish C3 deficient patient and 12 random, normal-intensity DBSS from our collection of control samples and tested for serum IgG levels. The intensities for all six low-intensity samples were comparable to those of the positive controls (data not shown), indicating that the samples were printed properly.

To investigate whether the six low-intensity samples contained substances that affected the results of the microarrays, the samples were mixed with normal-intensity DBSS eluates and C3 levels were retested. The results indicate that the low-intensity samples do not contain substances that affect the C3 analysis (data not shown).

Subsequently, the protein content in the six samples was determined by SDS-PAGE. A number of low molecular weight bands were noted, suggesting degradation of serum proteins (data not shown). No such bands were seen in the eluates from the C3 deficient patient or the normal-intensity controls, suggesting that C3 is one of the degraded proteins in these samples.

## Discussion

Today, a majority of PID patients are not diagnosed until brought to attention due to recurrent and/or severe infections. One of the reasons is the lack of high-throughput methods for analysis. Complement deficiencies represent one of the vastly under-diagnosed patient groups, with less than 10% of the affected individuals having been identified in Sweden [2]. In this study, we investigated whether C3 deficiency can be diagnosed in neonates by microarray analysis of DBSS. Based on our results, we show that C3 deficiency is present already at birth and it is possible to identify the deficient individuals both by microarrays and sandwich ELISA using DBSS eluates. Previously, we have used microarrays for detection of C3 in serum samples [19] but to our knowledge, this is the first study quantifying serum proteins eluted from DBSS using this approach.

The DBSS are normally collected within 72 hours after birth but can be stored for many years. The DBSS of the Swedish C3 deficient patient had been stored for 21 years prior to analysis. To exclude that the long-time storage may have resulted in degradation of the C3 protein, 13 DBSS stored for 8–23 years were included in the control group. As C3 was detected in all controls, we conclude that the lack of C3 in the patient sample is not a result of protein degradation. The undetectable C3 levels in the freshly collected serum sample from the C3 deficient patient also support this notion.

In the present study, higher concentrations of primary and secondary antibodies were required for microarray analysis of the DBSS than for the serum samples. As the composition of the DBSS eluates differs from that of the serum samples in terms of total protein content and viscosity, these parameters may be important for the piezo-electric printing procedure and might thus influence the amount of the sample that is actually transferred to the slide.

One of the limiting parameters in reverse phase microarrays is the theoretical sensitivity, originating from the small number of molecules transferred to the arrays during printing. If 0.4 nl of a complex sample, containing 1 g/l of a 180 kDa protein such as C3 (corresponding to 2.2 µM), is transferred to a slide, it will contain approximately 1.3×10^9^ molecules of this particular protein. Since only a fraction of the proteins present are available for detection, it is inherent in the technology that low-abundant proteins will be difficult to detect without proper signal amplification. With the present setup, the limit of detection for C3 in serum is approximately 500 µg/l, corresponding to 700 000 molecules. As the level of C3 in the working dilution of DBSS (1∶100) is approximately 10–20 mg/l, it is thus a suitable protein for analysis with microarrays.

As there is a great need for a high-throughput screening for PIDs, new approaches are currently being developed. In a recent study by Ingvarsson et al, 2007, sandwich antibody microarrays were used for multiplex analysis of eight complement proteins (including C3) in unfractioned serum and plasma [23], showing promising results for protein profiling in complex samples. However, the inherent format of the sandwich antibody arrays rules out the simultaneous screening of large numbers of samples that is required for population screening. Another approach for identification of immunodeficient patients was proposed by Chan & Puck in 2005, involving screening for severe combined immunodeficiency [22]. The method is based on quantification of T-cell receptor excision circles (TRECs) extracted from DBSS. The TRECs are generated during normal development of T cells but as the patients have very few, or no, T cells, no TRECs are produced. The authors detected TRECs in 232 out of 239 healthy controls but none in patients with severe combined immunodeficiency. The main drawback of this method is that it only identifies patients with severe combined immunodeficiency and some selected T cells disorders, comprising less than 1% of all patients with PID. Furthermore, the lack of detectable TRECs in the seven control samples probably represents a high rate of false positives.

We believe that a large-scale screening should ideally be based on the detection of one single analyte. As serum IgA levels are low or absent in most of the patients with various forms of both humoral and cellular PIDs, measurements of IgA levels in DBSS may theoretically provide a basis for a neonatal screening program. Another approach would be a multiplex array comprising of antisera for all known PIDs. However, the primary obstacle to such an approach is the present lack of suitable antisera for many of the proteins involved in these defects.

The microarray technique we have developed enables testing of thousands of samples simultaneously and the analysis can be extended to include additional analytes by increasing the number of slides used. Therefore, it represents an inexpensive and rapid method for analysis of serum proteins, and thus identifying patients with primary immunodeficiency lacking specific proteins.

## Materials and Methods

### Ethical statement

Approval for the study was obtained from the Regional Ethical Review Board in Stockholm. The approval applied to all the samples used in the work. Written consent was obtained when collecting patient DBSS. All other samples are anonymized.

### Collection of serum samples and DBSS

Serum samples from an adult Swedish C3 deficient patient [20] and twelve individuals with normal C3 levels were collected at the department of Clinical Chemistry, KUS Huddinge, Sweden. A serum sample from an adult Brazilian C3 deficient patient [21] was collected at Laboratory of Complement, Department of Immunology, Institute of Biomedical Sciences, University of São Paulo, São Paulo, Brazil and lyophilized. Before use this C3 deficient serum was reconstituted in 100 µl of PBS.

Neonatal DBSS from the Swedish C3 deficient patient (collected in 1985), 13 controls collected 1983–1998 and 256 freshly collected controls (2 dots, each 3 mm in diameter, corresponding to approximately 3 µl plasma) were obtained from the Center for Inherited Metabolic Diseases, Karolinska University Hospital Huddinge, Sweden. The 13 stored controls were included to investigate the impact of storage on the C3 levels.

### C3 levels in serum samples

Sandwich ELISA was performed for all serum samples using polyclonal rabbit anti-human C3c antibodies (DAKO, Denmark) at a final concentration of 3.8 µg/ml and horseradish peroxidase-conjugated polyclonal goat anti-human C3c antibodies (Nordic Immunological Laboratories, the Netherlands) at a final concentration of 3.3 µg/ml. The absorbance was read at 450 nm on a Vmax microplate reader (Molecular Devices, USA). For determination of C3 levels by microarrays, the serum samples were diluted 1∶100 in phosphate-buffered saline (PBS) with 0.5% Tween20 and printed onto Epoxide slides (Corning, the Netherlands) using a non-contact printing robot (Nano-plotter 2.0, Gesim, Germany), which deposits approximately 0.4 nl/drop. The slides were blocked with SuperBlock solution (Pierce Biotechnology, USA) and polyclonal rabbit anti-human C3c antibodies (DAKO, Denmark) were added at a final concentration of 115 ng/ml, followed by Alexa Fluor 555 conjugated goat anti-rabbit IgG antibodies (Molecular Probes, USA) at a final concentration of 33 ng/ml. The slides were scanned by a G2565BA array scanner (Agilent, Palo Alto, CA, USA) and the image analysis was performed with GenePixPro 5.1 (Axon Instruments, USA), using non-circular feature alignment.

### Preparation of simulated DBSS from the Brazilian C3D serum sample

A volume of 25 µl of adult blood from the Swedish C3D patient was centrifuged and added to 25 µl of the Brazilian serum sample. A volume of 25 µl (corresponding to ∼13 µl of plasma) of the mixed sample was then transferred onto filter paper and left to dry overnight. The filter paper was diluted 1∶50 in PBS with 0.5% Tween20 and soaked for one week at 4°C. The Swedish C3D serum sample and 0.9% NaCl, mixed with 25 µl of blood cells from the Swedish C3D patient, were used as negative controls and 9 serum samples with normal levels of C3 as positive controls. The samples were tested using ELISA and serum microarrays.

### C3 levels in DBSS

The DBSS from one Swedish C3 deficient patient, 256 freshly collected controls and 13 controls stored 8–23 years (containing approximately 3 µl of plasma), were soaked in 150 µl PBS with 0.5% Tween20, giving a final dilution of 1∶50. The DBSS were soaked for one week at 4°C and the C3 levels were determined by sandwich ELISA (as described above). For microarrays, the DBSS elutions were diluted to 1∶100. Polyclonal rabbit anti-human C3c antibodies (DAKO, Denmark) (2.3 µg/ml; 1∶500), and Alexa Fluor 555 conjugated goat anti-rabbit IgG antibodies (Molecular Probes, USA) (1 µg/ml; 1∶2,000) were used for detection. The correlation analyses were performed using Microsoft Excel.

### Serum IgG levels and total protein content of DBSS with low serum C3 levels

Serum IgG levels were determined by microarrays using polyclonal rabbit anti-human IgG (DAKO, Denmark) (83 ng/ml) and Alexa Fluor 555 conjugated goat-anti-rabbit IgG (Molecular Probes, USA) (33 ng/ml). An assessment of the protein content was made using a SDS-polyacrylamide gradient gel (NuPAGE 4–12% Bis-Tris pre cast gel, Invitrogen, USA). MultiMark rainbow molecular weight marker (Invitrogen, USA) was used as a standard and the gel was stained with Coomassie Brilliant Blue. The low-intensity DBSS were compared to eluates from DBSS of the Swedish C3 deficient patient and six random DBSS showing normal C3 levels on microarrays.
